# Estimation of drinking water volume of laboratory animals based on image processing

**DOI:** 10.1038/s41598-023-34460-w

**Published:** 2023-05-26

**Authors:** Zhihai Liu, Feiyi Liu, Qingliang Zeng, Xiang Yin, Yang Yang

**Affiliations:** 1 College of Transportation, Shandong University of Science and Technology, Qingdao, 266590 China; 2 College of Mechanical and Electronic Engineering, Shandong University of Science and Technology, Qingdao, 266590 China; 3 College of Information Science and Engineering, Shandong Normal University, Jinan, 250358 China

**Keywords:** Computer science, Mechanical engineering

## Abstract

This paper describes an image processing-based technique used to measure the volume of residual water in the drinking water bottle for the laboratory mouse. This technique uses a camera to capture the bottle's image and then processes the image to calculate the volume of water in the bottle. Firstly, the Grabcut method separates the foreground and background to avoid the influence of background on image feature extraction. Then Canny operator was used to detect the edge of the water bottle and the edge of the liquid surface. The cumulative probability Hough detection identified the water bottle edge line segment and the liquid surface line segment from the edge image. Finally, the spatial coordinate system is constructed, and the length of each line segment on the water bottle is calculated by using plane analytical geometry. Then the volume of water is calculated. By comparing image processing time, the pixel number of liquid level, and other indexes, the optimal illuminance and water bottle color were obtained. The experimental results show that the average deviation rate of this method is less than 5%, which significantly improves the accuracy and efficiency of measurement compared with traditional manual measurement.

## Introduction

With the development of medicine and biology, medical animal experiments have become essential to clinical medicine^1–3^. The demand for laboratory animal samples is also increasing. Therefore, more and more medical institutions and animal breeding institutions choose to raise experimental animals on a large scale. In breeding experimental animals, the most important thing is to meet the needs of animals for water and food. Experimental animals are usually kept in special cages, where they drink water from water bottles placed in the cages. When the remaining water in the water bottle in the feeding cage is insufficient, it is necessary to supplement it on time. Accurate and timely detection of residual water in water bottles is a prerequisite for ensuring the drinking water needs of experimental animals.

Designing measurement schemes through various sensors is the most commonly used method for measuring the volume of water in containers. However, the narrow cage space and the frequent activity of animals in the cage will limit the installation of sensors, making it difficult to measure the remaining amount of water and food by installing sensors. In the existing feeding mode, the method of visually observing the water bottle by the breeder is usually selected to estimate the water amount roughly. Artificial methods require a lot of energy and time for breeders, but frequent personnel entry and exit may also damage the sterile growth environment necessary for experimental animals. In addition, artificial methods also limit the improvement of automation in the feeding process. Therefore, proposing a non-contact detection method for water residue in water bottles is significant.

Scholars in various fields have researched automated measurement methods for container liquid volume and proposed many solutions. Singh et al.^4^ proposed a liquid volume measurement method based on optical fiber sensors. Since the deformation degree of the fiber ring in the sensor is proportional to the liquid volume in the container, the liquid volume in the container can be measured by detecting the data fed back by the sensor. O. Alonso Hernandez et al.^5^ proposed a measurement system for residual oil volume in a fuel tank based on an infrared transmitter and receiver. This method measures the distance between the liquid level in the container and the receiver based on the intensity of the infrared signal received by the receiver by installing a float in the liquid container and then calculating the volume of the liquid. Zhang Jingyue et al.^6^ proposed a liquid level measurement method based on laser and machine vision technology to measure liquid volume in glass gauges. By analyzing the refraction phenomenon of laser light in the liquid to be measured, the corresponding liquid volume value can be obtained by calculating the liquid level height. Parisa Esmaili et al.^7^ proposed a detection scheme for container liquid levels based on pressure sensors. This scheme collects the pressure generated by the liquid in the container through sensors installed inside the container and then calculates the volume of the liquid. Kazuyuki Kobayashi et al.^8^ proposed a Doppler module-based method for container liquid level detection. This method judges the position of the liquid level line based on the different reflection characteristics of microwave waves on various materials. It can then obtain the volume of water in the container. Z. Zakaria et al.^9^ proposed a detection method based on ultrasonic sensors, which judges whether there is liquid at the detection location based on the differences in the propagation of ultrasonic waves in different media, and then infers the position of the liquid level line. Shaheen Ahmad et al.^10^ proposed a liquid level detection method based on capacitive sensors, which can reflect the changes in liquid level in the form of electrical capacity changes, thereby realizing the detection of liquid level position. Analyze the principles of the above methods and summarize the advantages and disadvantages of each method in Table 1.

**Table 1:** The measurement principle, advantages and disadvantages of the above methods.

Measuring method	Measuring principle	Advantages	Disadvantages
Optical fiber sensors	Measuring the pressure gener-ated by the liquid through an optical fiber sensor to calcula-te the volume of the liquid	There are no restrictions on the type of liquid to be measured	Need supporting signal processing devices with complex structure
Infrared transmitter	The camera cooperates with the laser transmitter to obtain the liquid level line position	Lower cost compared to alternatives	Not suitable for small volume containers
Laser transmitter	Calculate the distance betwe-en the infrared emitter and the liquid level to calculate the volume of the liquid	Experimental samples with less liquid volume have higher measurement accu-racy, non-contact detection	Only the volume of liquid in horizontally placed containers can be measured
Pressure sensors	Calculate the liquid volume using the liquid pressure measured by the pressure sensor	The method can measure the volume of liquid in the oscillation	The measurement system has a complex structure and is not suitable for use in animal feeding enviro- nments
Doppler module	Differences in microwave reflection characteristics for different materials	Non contact detection, the measurement results are robust to changes in temperature and conductiv-ity	The microwave transmit-ter needs to be installed in the optimal location, resulting in many restrictions in use
Ultrasonic sensors	Differences in propagation characteristics of acoustic waves to different materials	Non-contact measurement, no restriction on the type of liquid to be measured	Not suitable for small volume containers with low measurement accur-ary
Capacitive sensors	Convert liquid level changes into capacitance changes	High detection sensitivity and timeliness	The measurement system is too complex

After thoroughly studying the feeding environment and living habits of medical animals, it is concluded that the following characteristics are required for the measurement method of liquid volume in bottles used in this scenario: 1. The measurement method needs to be timely in order to determine whether the water in the bottle needs to be replenished on time; The measurement method should avoid affecting the expected growth of medical animals; Due to the small space of the feeding cage, the measurement method should have a relatively simple hardware structure; 4. The measurement method should have a good economy while ensuring accuracy.Summarizing the principles, advantages, and disadvantages of the above liquid volume measurement algorithms, the current mainstream detection schemes are mainly based on various sensors with relatively complex structures and high costs. They are not suitable for application in medical animal feeding scenarios. In addition, the installation location of the sensor too close to the animal being raised can also pose a potential hazard.

Machine vision is a technology involving sensors that simulate the human eyes collecting and processing images. By detecting the target image's position, shape, texture, and other characteristics, parameters such as object size can be quantified. Therefore, machine vision technology can replace manual measurement of residual water in water bottles^11–17^. Based on the specific needs of medical animal feeding scenarios, this paper proposes a method for detecting the remaining water volume in water bottles based on machine vision. In our research, edge detection algorithms, image segmentation algorithms, and line detection algorithms are used to process the captured water bottle image to obtain key feature parameters and calculate the volume of water in the water bottle. The experiment used the most common mice-feeding cage in medical animal feeding environments as an example. This method can achieve real-time non-contact detection of the volume of water in the bottle, avoiding adverse effects on the growth of medical animals. This detection method only requires light sources, cameras, and computers, without adding additional sensors. It has a simple structure and is easy to install, ensuring high measurement accuracy and good economy.

## Materials and methods

### Experimental setup and data collection

Experiments were conducted in December 2019 at Shandong University of Science and Technology, Qingdao, Shandong Province, China. The bottles were purchased from the breeding grounds of laboratory mice. The experiment was carried out at room temperature of 25℃. During the experiment, in order to reduce the error generated during the transfer of water from the measuring cylinder to the water bottle, the mass of water in each water bottle is determined by weighing with a calibrated electronic scale, and the volume in the bottle is calculated using the previously measured density. The image processing algorithms involved in this paper are all based on opencv 4.4.0.

**Figure 1:**
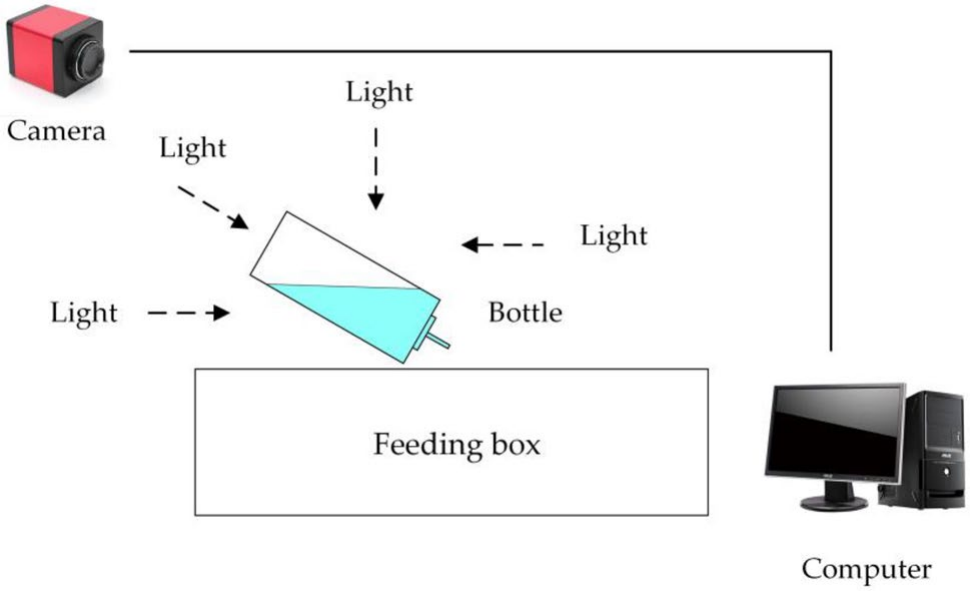
Experimental equipment and image acquisition system.

Figure 1 shows the image acquisition system composed of a CMOS camera, light source, support frame, and computer. The resolution of the camera is 1920 pixels × 1080 pixels. The camera captured the water bottle at a horizontal angle of 45° from a high angle of 45°. The light source was in line with the camera and the water bottle to illuminate and enhance the contrast between the liquid level and the background. Sixty-eight water groups of different quality were weighed and bottled separately and placed in a laboratory mice incubator for images collection. The resolutions of the acquired images were adjusted to be 1344 pixels × 756 pixels to meet the actual detection speed.

### Image acquisition system

**Figure 2:**
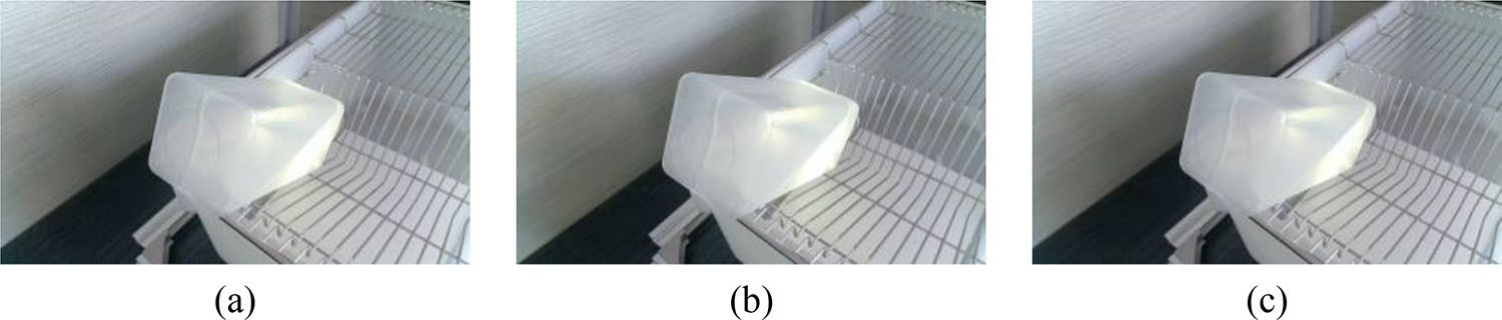
Images of different volumes water stored in the same water bottle (white bottle): (**a**) 75 ml; (**b**) 80 ml and (**c**) 85 ml.

**Figure 3:**
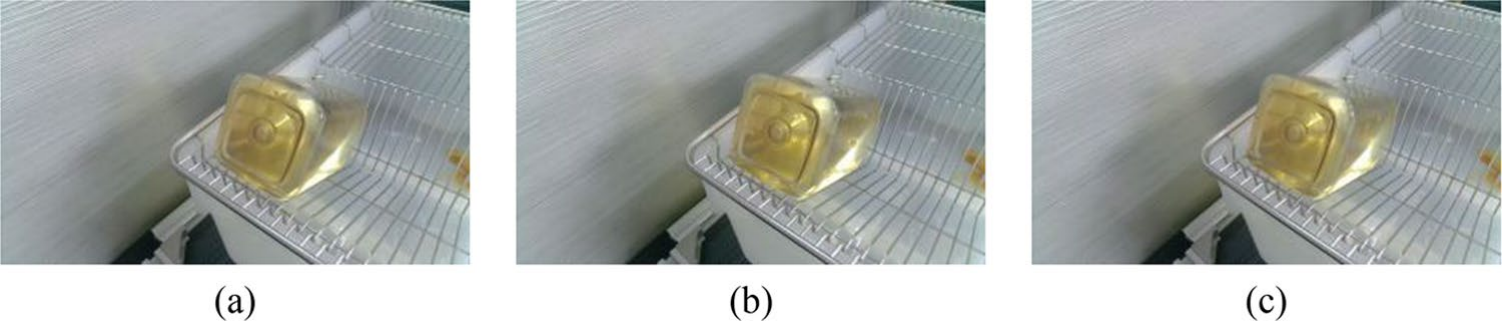
Images of different volumes water stored in the same water bottle (brown bottle): (**a**) 130 ml; (**b**) 140 ml and (**c**) 150 ml.

**Figure 4:**
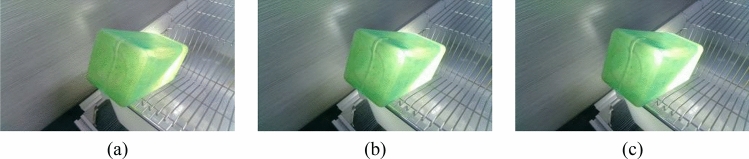
Images of different illuminance stored in the same water bottle (green bottle): (**a**) 91 lx; (**b**) 116 lx and (**c**) 140 lx.

**Figure 5:**
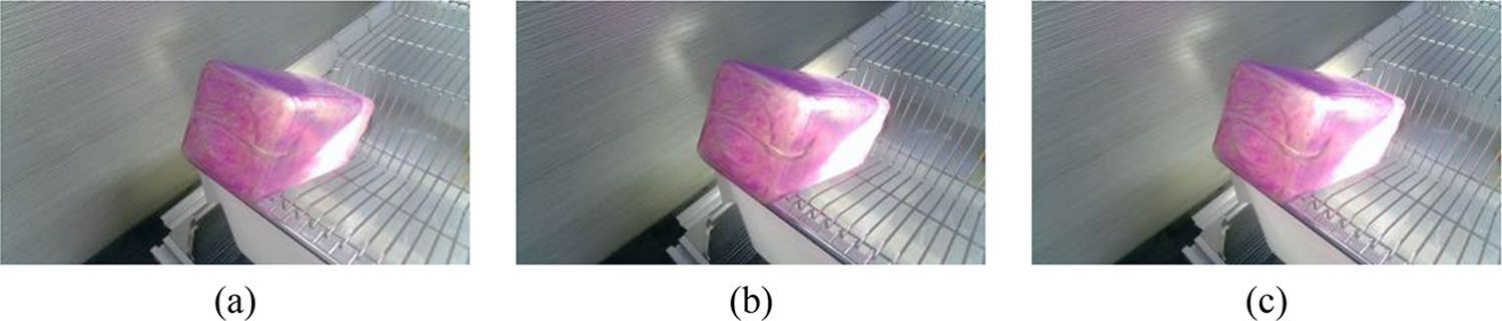
Images of different illuminance stored in the same water bottle (purple bottle): (**a**) 91 lx; (**b**) 116 lx and (**c**) 140 lx.

The imaging system consisted of an HD camera (SY003-V01, WeiXinShiJie, Wuhan, China) mounted on a metal stand at 0.3 m(within the field of view) above the box shown in Fig. 1. The HD camera was connected via a USB port to a computer installed with an Intel i5- 7200U CPU, 2.5 GHz, 8 GB physical memory, Microsoft Windows 10 operation system, and Software Development Kit (SDK). Images were acquired from the HD camera using Visual Studio 2017 software with the image acquisition Toolkit. The images involved in this paper are all RGB images. The images were transferred to a 1 TB hard drive for subsequent analysis. Images are captured from each water bottle and shown in Figs. 2–5.

**Figure 6:**
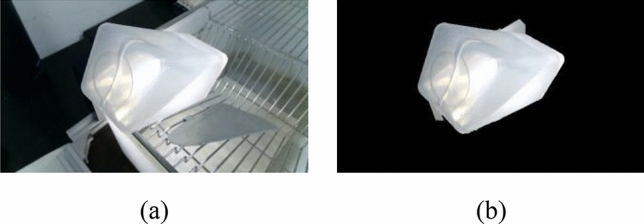
Foreground image based on Grabcut algorithm (white bottle): (**a**) original image and (**b**) segmented image.

There are two mainstream laboratory mice drinking bottles on the market, as shown in Fig. 3 and 4. Figure 3 is white translucent bottles, and Fig. 4 is brown transparent bottles. Two different color water bottles were made for the experiment more rigorous to determine the influence of varying color bottles on the measurement speed and light source on the judgment of liquid level position of different color bottles. Figure 5 is green translucent bottles, and Fig. 6 is purple.

### Foreground extraction

The Grabcut image segmentation algorithm was mainly used for the water bottle foreground extraction. The Grabcut algorithm is an improved algorithm based on the Graphcut algorithm.^18–25^ Through the Grabcut algorithm, the water bottle can be separated from the background to obtain a foreground target and processed.

In the foreground extraction process, the foreground area of the water bottle image is first annotated with a rectangular box. Due to the fixed shooting position of the image, the position of the water bottle as the foreground in the image is also fixed, so the position of the rectangular box is a predetermined unified value.

Create a K-dimensional full-covariance Gaussian Mixture Model (GMM) to process the pixels of the foreground and the background of the water bottle, These models will be used to determine the foreground or background probability of each pixel in the image. Using classification information for iterative segmentation, in each iteration, the algorithm updates the energy function using the classification results from the GMM, and performs segmentation by maximizing the energy function. The energy function involved in iterative segmentation is as follow:

1 $$\begin{array}{c}E\left(\alpha ,k,\theta ,x\right)=U\left(\alpha ,k,\theta ,x\right)+V\left(\alpha ,x\right)\end{array}$$

In the formula, *E* is the Grabcut energy function, and the smaller its value, the better the image segmentation effect; *U* is the region term, indicating the probability of assigning pixels to the foreground or background; *V* is the smoothing term, indicating the similarity between adjacent pixels; α is opacity, α $\in$ [0,1], 0 is the image's background, and 1 is in the foreground of the image; *k* represents the *k*-th Gaussian component in the GMM model;* x* is the input image. θ is a parameter in GMM, which is a set of mean vectors, covariance matrices, and weights, θ can be defined as:

2 $$\begin{array}{c}\theta =\left\{\pi \left(\alpha ,k\right),\mu \left(\alpha ,k\right),\sum \left(\alpha ,k\right),\alpha =\mathrm{0,1};k=\mathrm{1,2},3\dots ,k\right\}\end{array}$$

where π (α, *k*) is the combined weight of each Gaussian component; μ (α, *k*) is the mean of each Gaussian model; $\sum \left(\alpha ,k\right)$ is the covariance matrix. The parametric model performs an iterative operation. After each iteration, the current segmentation result is converted into a new image and used as the initial state of the next iteration until the iterative process converges and reaches an optimal state. After the iteration is completed, set the pixel value of the background part of the image to 0 and output the extraction result of the water bottle in the image. Take the white water bottle as an example, Fig. 6a is the original image, and Fig. 6b is the segmented image.

### Edge detection

The surface of the water bottle has irregular textures, which will affect the detection effect of the algorithm on the edge line and liquid level line of the water bottle. Use Canny edge detection operator to eliminate the influence of bottle texture and improve features such as level and edge^26–30^.a. Gaussian filtering

The noise of the image affects the processing of the image, so the noise is preferentially filtered to prevent false detections. The image is convolved using a Gaussian filter to smooth the image, reducing the apparent noise effects on the edges.b. Calculate the gradient amplitude and direction

The edges can point in different directions. Use the edge difference operator to calculate the horizontal difference ${G}_{x}$ and the vertical difference ${G}_{y}$ as:

3 $$\begin{array}{c}\left\{\begin{array}{c}{G}_{x}\left(i,j\right)=\frac{1}{2}\left[M\left(i+1,j\right)-M\left(i,j\right)+M\left(i+1,j+1\right)-M\left(i,j+1\right)\right]\\ {G}_{y}\left(i,j\right)=\frac{1}{2}\left[M\left(i,j+1\right)-M\left(i,j\right)+M\left(i+1,j+1\right)-M\left(i+1,j\right)\right]\end{array}\right.\#\end{array}$$

where $M\left(i,j\right)$ is the gray value of the pixel of the gray image.

The modulus $G\left(i,j\right)$ of the gradient is:

4 $$\begin{array}{c}G\left(i,j\right)=\sqrt{{G}_{x}^{2}\left(i,j\right)+{G}_{y}^{2}\left(i,j\right)}\end{array}$$

**Figure 7:**
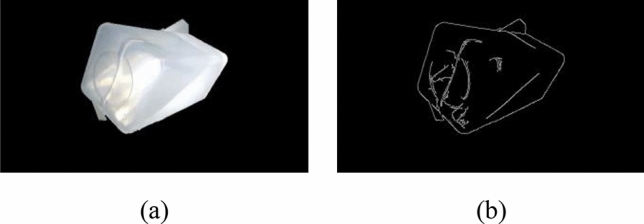
Edges detected by the Canny operator: (**a**) foreground image based on Grabcut algorithm and (**b**) the image obtained by Canny operator.

The gradient direction $\theta \left(i,j\right)$ is:

5 $$\begin{array}{c}\theta \left(i,j\right)=arctan\left[\frac{{G}_{x}\left(i,j\right)}{{G}_{y}\left(i,j\right)}\right]\end{array}$$

c. Non-maximum suppression:

The gradient intensity of the current point is compared to that of the positive and negative gradient direction points. Retain the gradient strength of the current point if it is significant enough. Otherwise, the suppression is set to 0. The effect of non-maximum suppression is to refine the edges of the original blur.d. Double threshold:

The Canny algorithm detects the gradient value of boundary points and applies high and low thresholds to distinguish edge pixels. Set a high threshold *H* with a low threshold *L*. If $G(i,j)$ > *H*, it means that $G(i,j)$ is regarded as a strong edge point; if *H* ≤ $G(i,j)$  ≤ *L*, it means that $G(i,j)$ is regarded as a weak edge point; If $G(i,j)$< *L*, it means that the point $G(i,j)$ is suppressed. After edge detection, strong edge points are determined to be the edge part of the image, while weak edge points are determined to be the edge part of the image based on whether they are adjacent to the strong edge. Because the position of the liquid level in the image belongs to the weak edge, to ensure that the liquid level position can be recognized in the later image processing, this paper reduces the threshold in the aspect of edge detection to ensure the accuracy of the liquid level position. In this paper, the high threshold is set to 120, the low threshold is set to 80, and the core size is set to 3 × 3. Both vertical and horizontal standard deviations are 0.8.Fig. 7a is the segmented image, and Fig. 7b is the image obtained by the Canny operator.

Because the position of the liquid level in the image belongs to the weak edge, to ensure that the liquid level position can be recognized in the later image processing, this paper reduces the threshold in the aspect of edge detection to ensure the accuracy of the liquid level position. Figure 7a is the segmented image, and Fig. 7b is the image obtained by the Canny operator.

### Line detection

It is necessary to obtain parameters such as the edge of the water bottle, the edge of the liquid surface, and the inclination angle of the water bottle to calculate the volume of water in the water bottle. In this paper, the cumulative probability Hough transform^31–34^ is used to obtain key line segment's expression and the line segment's endpoint. Mark the pixel points in the extracted edge image, generate a set of lines passing through the point at different angles, and represent the set in polar coordinates. The mathematical expression is as follows:

6 $$\begin{array}{c}r=x\mathit{cos}\theta +y\mathit{cos}\theta \end{array}$$

In the equation, $\left(x,\mathrm{y}\right)$ is the coordinate of a point on the line, $r$ is the shortest distance from the origin to the line where the point $\left(x,\mathrm{y}\right)$ is located; *θ* is the angle between the line equation of r and the x-axis.

**Figure 8:**
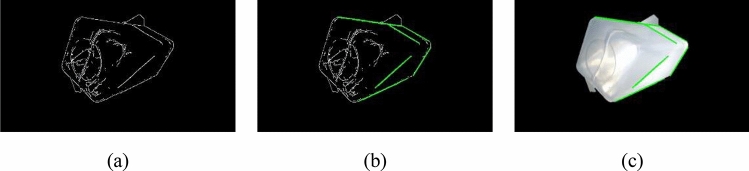
Probabilistic Hough Transform: (**a**) the image obtained by the Canny operator (white bottle) , (**b**) the straight line obtained by the probability Hough transform and (**c**) Schematic diagram of the position of the extracted line on the water bottle.

The minimum voting threshold in this paper is set to 100. When the intersection points between curves exceed the minimum voting threshold, it is determined that the points forming these curves belong to the same line in the Cartesian coordinate system, and all lines in the image can be extracted by traversing all curves in the polar coordinate system. By filtering the extracted straight lines through a fixed slope range, the liquid level line and key contour line of the water bottle can be obtained. In order to more intuitively understand the effect of the algorithm, the detected straight line is marked on the water bottle. As shown in Fig. 8, according to needs, this paper selects straight lines representing the water bottle container's length, width, height, and liquid level respectively for calculation. The straight-line extracted by this method is relatively complete and has no fracture, which can meet the requirements of the following analysis.

### Balance calculation

**Figure 9:**
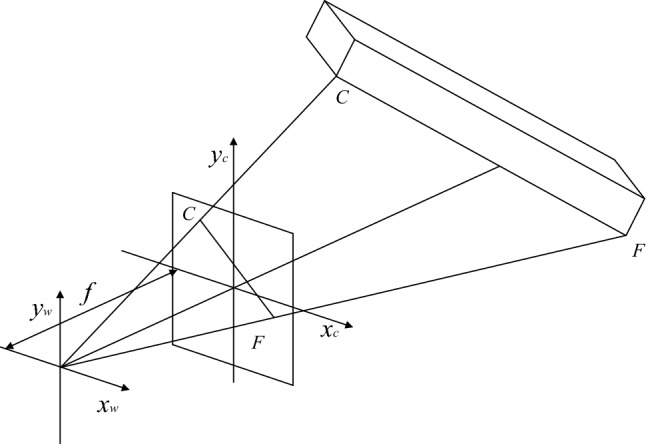
The principle of camera coordinate system projection.

In the actual situation, the relative position of the water bottle and the camera remains the same, so projection and perspective are used to obtain the data of the water bottle. Use the projection length ${L}_{x}$ of each straight line of the water bottle body and the proportional relations $\alpha$ of the perspective to calculate the volume of liquid. Because different sides of the water bottle have different positions, the projections’ length and perspective ratio are also different. In this research, take one of the edges as an example. The schematic is shown in Fig. 9. o-${x}_{w}$-${y}_{w}$-${z}_{w}$ and o-${x}_{c}$-${y}_{c}$-${z}_{c}$ are the world coordinate and camera coordinate systems, respectively. $FC$ is a sideline of the water bottle, ${F}^{\mathrm{^{\prime}}\mathrm{^{\prime}}}{C}^{\mathrm{^{\prime}}\mathrm{^{\prime}}}$ is the projection of $FC$ in the camera coordinate system o-${x}_{c}$-${y}_{c}$-${z}_{c}$, and $f$ is the camera focal length.

**Figure 10:**
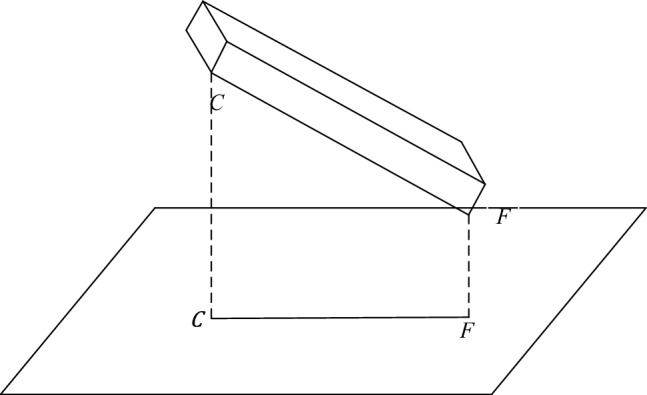
Principles of Plane projection system.

According to the projection principle, the projection of the line segment $FC$ is ${{F}^{\mathrm{^{\prime}}}C}^{\mathrm{^{\prime}}}$, as shown in Fig. 10. The ratio ${\alpha }_{FC}$ of the length of line segment $FC$ to line segment ${{F}^{\mathrm{^{\prime}}}C}^{\mathrm{^{\prime}}}$ is

7 $$\begin{array}{c}{\alpha }_{FC}=\frac{{L}_{FC}}{{L}_{{F}^{\mathrm{^{\prime}}}{C}^{\mathrm{^{\prime}}}}}\end{array}$$

**Figure 11:**
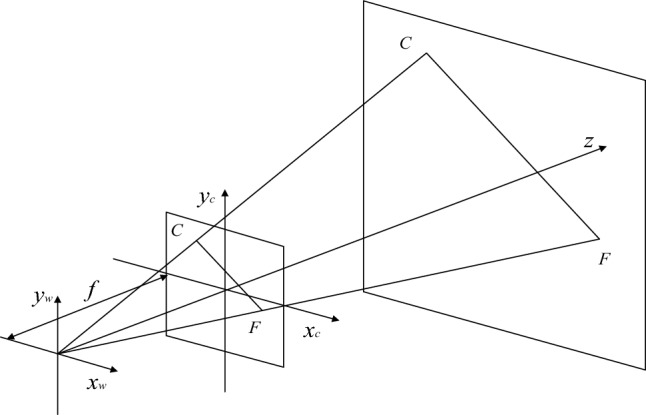
Schematic of perspective.

As shown in Fig. 11, The proportional relationship between the projection plane and the imaging plane is obtained by the perspective principle

8 $$\begin{array}{c}{\beta }_{FC}=\frac{{L}_{{F}^{\mathrm{^{\prime}}}{C}^{\mathrm{^{\prime}}}}}{{L}_{{F}^{\mathrm{^{\prime}}\mathrm{^{\prime}}}{C}^{\mathrm{^{\prime}}\mathrm{^{\prime}}}}}\end{array}$$

**Figure 12:**
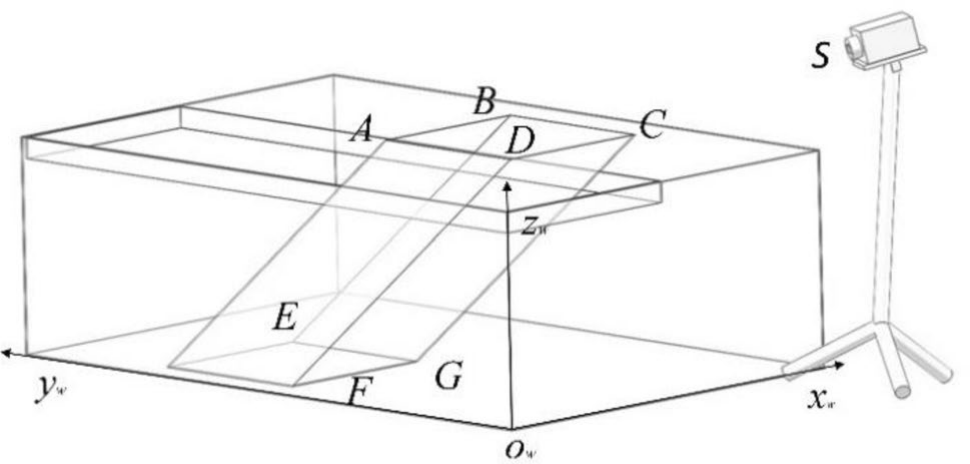
World coordinate system and camera position.

The setting of the world coordinate system is shown in Fig. 12. The measurement algorithm proposed in this paper can calculate the volume of liquid in the bottle based on the edge line and the position of the liquid level line of the water bottle in the image. On the premise of ensuring the integrity of the liquid level line on the bottle in the captured image, the camera position and shooting angle will not change the principle and results of the detection algorithm. In this paper, the camera coordinates are $\left({x}_{s},0,{z}_{s}\right)$, The shooting angle is 45 degrees in the horizontal direction and 45 degrees in the vertical direction. In this case, the expression of the plane S where the camera is located is as follows:

9 $$\begin{array}{c}x-y+z-{x}_{s}-{z}_{s}=0\end{array}$$

Then the normal vector of the plane $S$ is $\overrightarrow{n}=\left(1,-\mathrm{1,1}\right)$. The coordinates of each point on the image and the expression of each edge in the bottle body can be obtained by the advance measurement.

Take one of the edges $FC$ as an example, and set the vector $\overrightarrow{FC}=\left({x}_{C}-{x}_{F},{{y}_{C}-y}_{F},{{z}_{C}-z}_{F}\right)$, the cosine of the vector $\overrightarrow{FC}$ and the normal vector $\overrightarrow{n}$ is:

10 $$\begin{array}{c}\mathit{cos}\theta =\frac{\overrightarrow{n}*\overrightarrow{FC}}{\left|\overrightarrow{n}\right|*\left|\overrightarrow{FC}\right|}=\frac{{x}_{C}-{x}_{F}-{{y}_{C}+y}_{F}+{{z}_{C}-z}_{F}}{\sqrt{3}*\sqrt{{\left({x}_{C}-{x}_{F}\right)}^{2}+{\left({{y}_{C}-y}_{F}\right)}^{2}+{\left({{z}_{C}-z}_{F}\right)}^{2}}}\end{array}$$

Then the projection of the vector $\overrightarrow{FC}$ on the plane is:

11 $$\begin{array}{c}{L}_{{F}^{\mathrm{^{\prime}}}{C}^{\mathrm{^{\prime}}}}={L}_{FC}*\sqrt{1-{\left(\mathit{cos}\theta \right)}^{2}}\end{array}$$

where ${L}_{FC}$ is the measured length of the known line segment.

Therefore, the ratio $\alpha$ between the actual length of the line segment and the projected length is:

12 $$\begin{array}{c}{\alpha }_{FC}=\frac{{L}_{FC}}{{L}_{{F}^{\mathrm{^{\prime}}}{C}^{\mathrm{^{\prime}}}}}\end{array}$$

The ratio of the projection length of each line segment to the length of the image $\beta$ is:

13 $$\begin{array}{c}{\beta }_{FC}=\frac{{L}_{{F}^{\mathrm{^{\prime}}}{C}^{\mathrm{^{\prime}}}}}{{L}_{{F}^{\mathrm{^{\prime}}\mathrm{^{\prime}}}{C}^{\mathrm{^{\prime}}\mathrm{^{\prime}}}}}\end{array}$$

where ${L}_{{F}^{\mathrm{^{\prime}}\mathrm{^{\prime}}}{C}^{\mathrm{^{\prime}}\mathrm{^{\prime}}}}$ is the length of the line segment on the graph.

Then the formula for changing the length of the line segment on the image to the length of the corresponding actual line segment is:

14 $$\begin{array}{c}{L}_{x}={\alpha }_{x}*{\beta }_{x}*{L}_{{x}^{\mathrm{^{\prime}}\mathrm{^{\prime}}}} \end{array}$$

where $x$ is the corresponding line segment.

**Figure 13:**
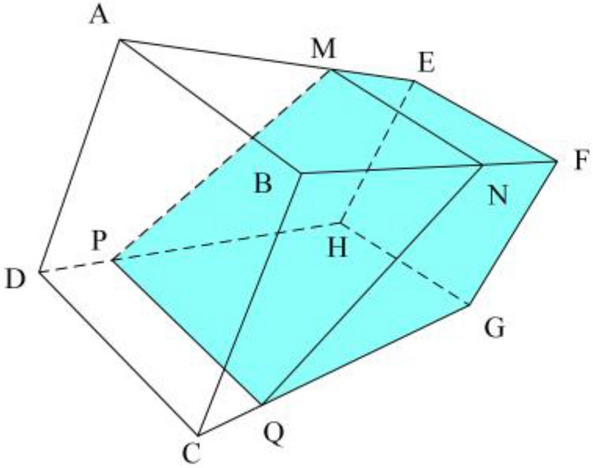
Complement edge line(a medium amount of water).

To facilitate the calculation, extract and complete the edge lines, as shown in Fig. 13. Since the water bottle is inserted obliquely on the laboratory mice incubator, and the bottleneck is irregular, the total volume of the water bottle minus the volume of the air can be used to obtain the volume of water in the water bottle. Since the existing inclination angle is known as $\gamma$, the length of each line on the image is:

15 $$\begin{array}{c}\left\{\begin{array}{c}\begin{array}{c}{L}_{FG}=\sqrt{{\left({x}_{F}-{x}_{G}\right)}^{2}+{\left({y}_{F}-{y}_{G}\right)}^{2}}{*\alpha }_{FG}*{\beta }_{FG}\\ {L}_{EF}=\sqrt{{\left({x}_{E}-{x}_{F}\right)}^{2}+{\left({y}_{E}-{y}_{F}\right)}^{2}}{*\alpha }_{EF}*{\beta }_{EF}\end{array}\\ {L}_{QG}=\sqrt{{\left({x}_{Q}-{x}_{G}\right)}^{2}+{\left({y}_{Q}-{y}_{G}\right)}^{2}}{*\alpha }_{CG}*{\beta }_{CG}\#\#\#\\ {L}_{FN}=\sqrt{{\left({x}_{F}-{x}_{N}\right)}^{2}+{\left({y}_{F}-{y}_{N}\right)}^{2}}{*\alpha }_{BF}*{\beta }_{BF}\end{array}\right.\end{array}$$

16 $$V_{a} = \frac{1}{6}L_{FG} *\left[ {L_{EF} {*}L_{QG} + L_{EF} {*}L_{FN} + 2\left( {L_{FN} + L_{QG} } \right){*}L_{EF} } \right]$$

**Figure 14:**
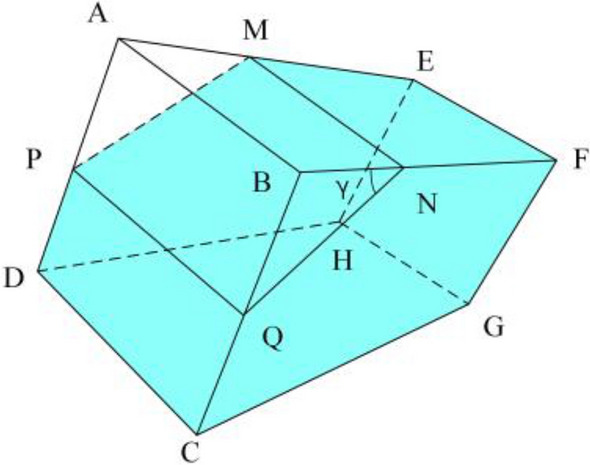
Complement edge line(a large amount of water).

The air volume calculation formula is different for water bottles containing different amounts of water. By detecting the position of the liquid level line $MN$, select the appropriate calculation formula. As shown in Fig. 14, when there is a large amount of water in the bottle, the formula for calculating the volume of air in the bottle can be obtained.

17 $$\begin{array}{c}\left\{\begin{array}{c}{L}_{BQ}=\sqrt{{\left({x}_{B}-{x}_{Q}\right)}^{2}+{\left({y}_{B}-{y}_{Q}\right)}^{2}}{*\alpha }_{BC}*{\beta }_{BC}\\ {L}_{BN}=\sqrt{{\left({x}_{B}-{x}_{N}\right)}^{2}+{\left({y}_{B}-{y}_{N}\right)}^{2}}{*\alpha }_{BF}*{\beta }_{BF}\\ {L}_{AB}=\sqrt{{\left({x}_{A}-{x}_{B}\right)}^{2}+{\left({y}_{A}-{y}_{B}\right)}^{2}}{*\alpha }_{AB}*{\beta }_{AB}\end{array}\right.\end{array}$$

18 $$\begin{array}{c}{V}_{a}=\frac{1}{2}{L}_{BQ}*{L}_{BN}*{L}_{AB}\end{array}$$

**Figure 15:**
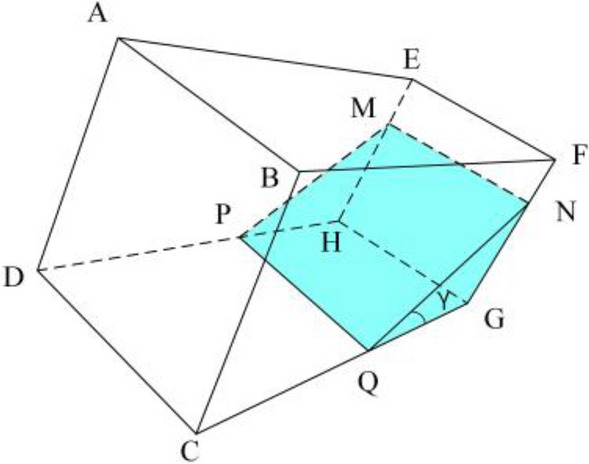
Complement edge line(a small amount of water).

As shown in Fig. 15, when there is only a small amount of water in the bottle, the formula for calculating the air volume in the bottle can be obtained.

19 $$\begin{array}{c}\left\{\begin{array}{c}\begin{array}{c}{L}_{CQ}=\sqrt{{\left({x}_{C}-{x}_{Q}\right)}^{2}+{\left({y}_{C}-{y}_{Q}\right)}^{2}}{*\alpha }_{CG}*{\beta }_{CG}\\ {L}_{EF}=\sqrt{{\left({x}_{E}-{x}_{F}\right)}^{2}+{\left({y}_{E}-{y}_{F}\right)}^{2}}{*\alpha }_{EF}*{\beta }_{EF}\end{array}\\ \begin{array}{c}{L}_{BF}=\sqrt{{\left({x}_{B}-{x}_{F}\right)}^{2}+{\left({y}_{B}-{y}_{F}\right)}^{2}}{*\alpha }_{BF}*{\beta }_{BF}\\ {L}_{FN}=\sqrt{{\left({x}_{F}-{x}_{N}\right)}^{2}+{\left({y}_{F}-{y}_{N}\right)}^{2}}{*\alpha }_{FG}*{\beta }_{FG}\end{array}\\ {L}_{GN}=\sqrt{{\left({x}_{G}-{x}_{N}\right)}^{2}+{\left({y}_{G}-{y}_{N}\right)}^{2}}{*\alpha }_{FG}*{\beta }_{FG}\end{array}\right.\end{array}$$

20 $$\begin{array}{c}{V}_{a}=\frac{1}{6}{L}_{GN}*\left[{L}_{CQ}*{L}_{EF}+{L}_{BF}*{L}_{EF}+2\left({L}_{BF}+{L}_{CQ}\right){*L}_{EF}\right]+{L}_{FN}*{L}_{BF}*{L}_{EF}\#\end{array}$$

Then the remaining volume of water ${V}_{w}$ is:

21 $$\begin{array}{c}{V}_{w} = V-{V}_{a}\end{array}$$

In the formula: $V$ is the fixed capacity of a bottle.

## Results and discussion

### Influence of light conditions and water bottle color

**Figure 16:**
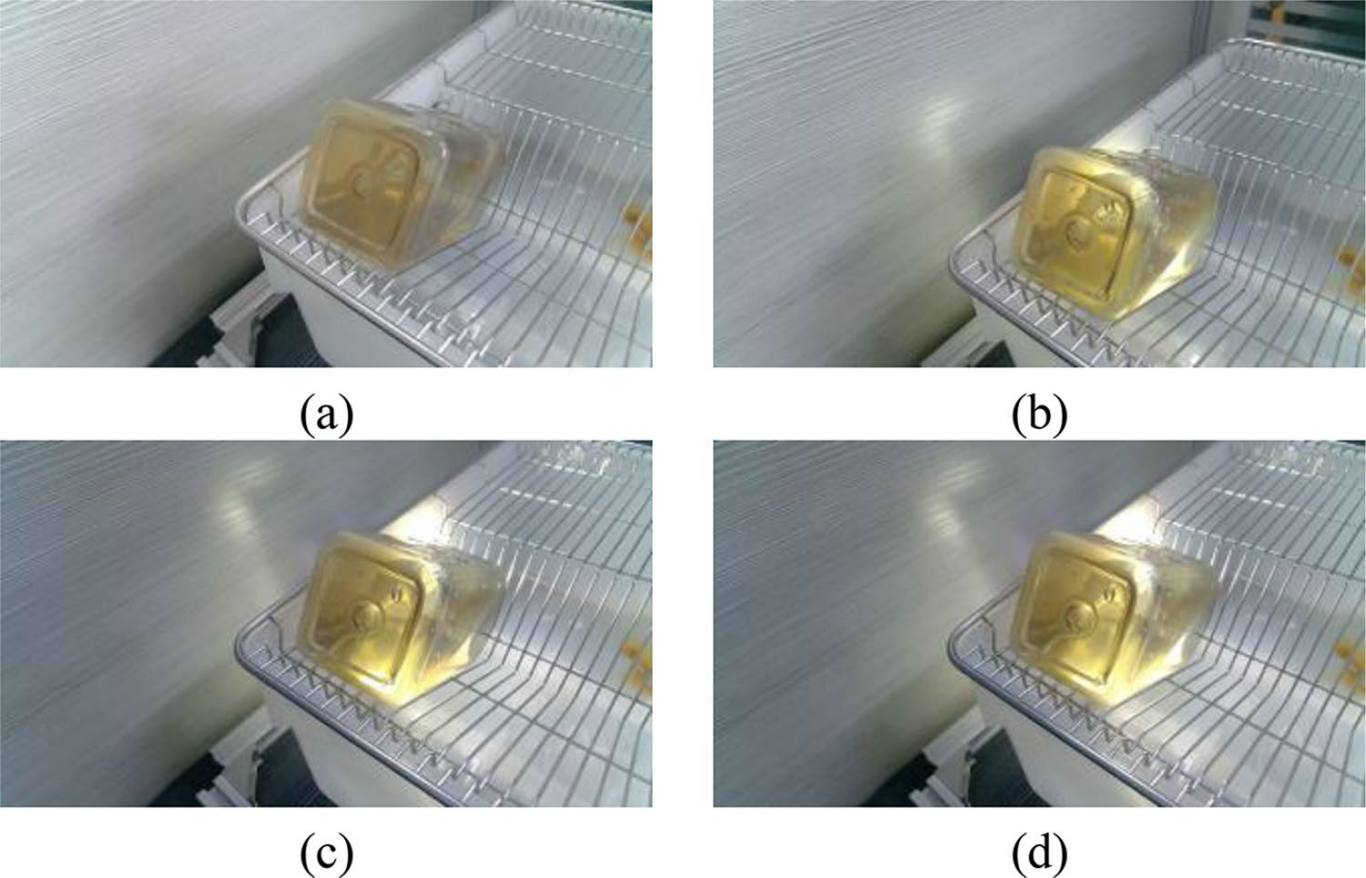
Bottles in different Illuminances (brown bottle): (**a**) 75 lx, (**b**) 91 lx, (**c**) 116 lx and (**d**) 140 lx.

**Figure 17:**
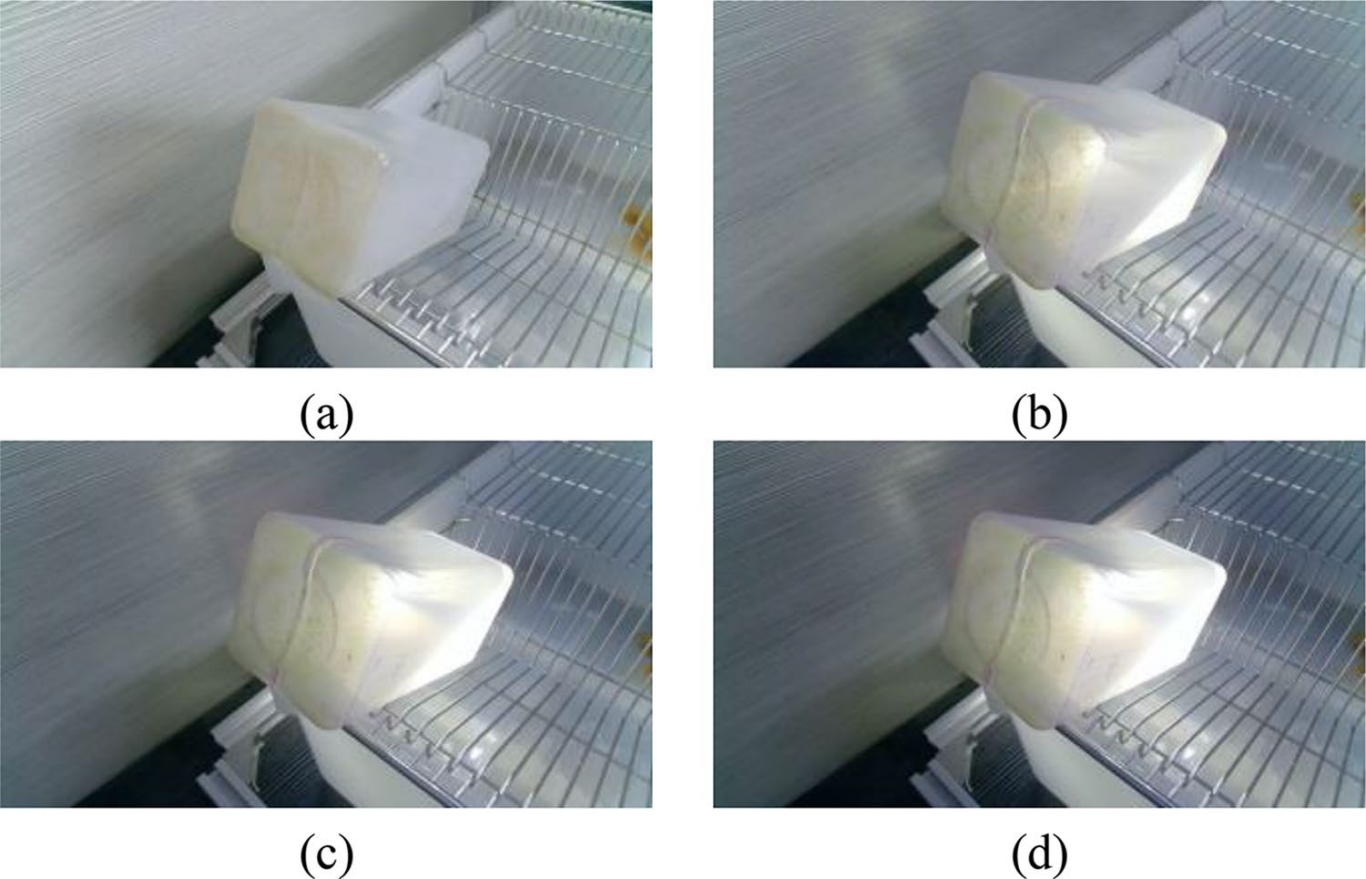
Bottles in different Illuminances (white bottle): (**a**) 75 lx, (**b**) 91 lx, (**c**) 116 lx and (**d**) 140 lx.

**Figure 18:**
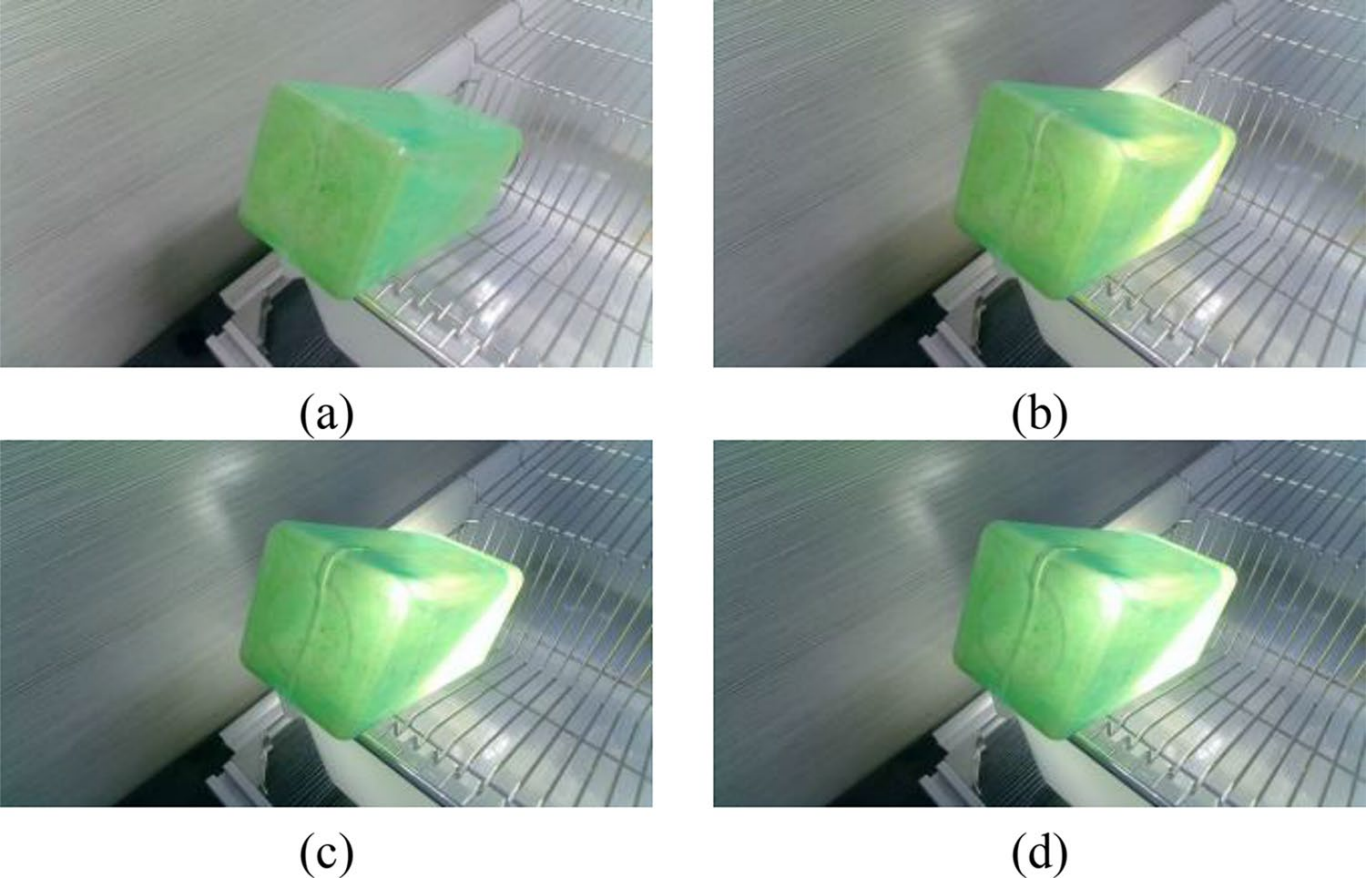
Bottles in different Illuminances (green bottle): (**a**) 75 lx, (**b**) 91 lx, (**c**) 116 lx and (**d**) 140 lx.

**Figure 19:**
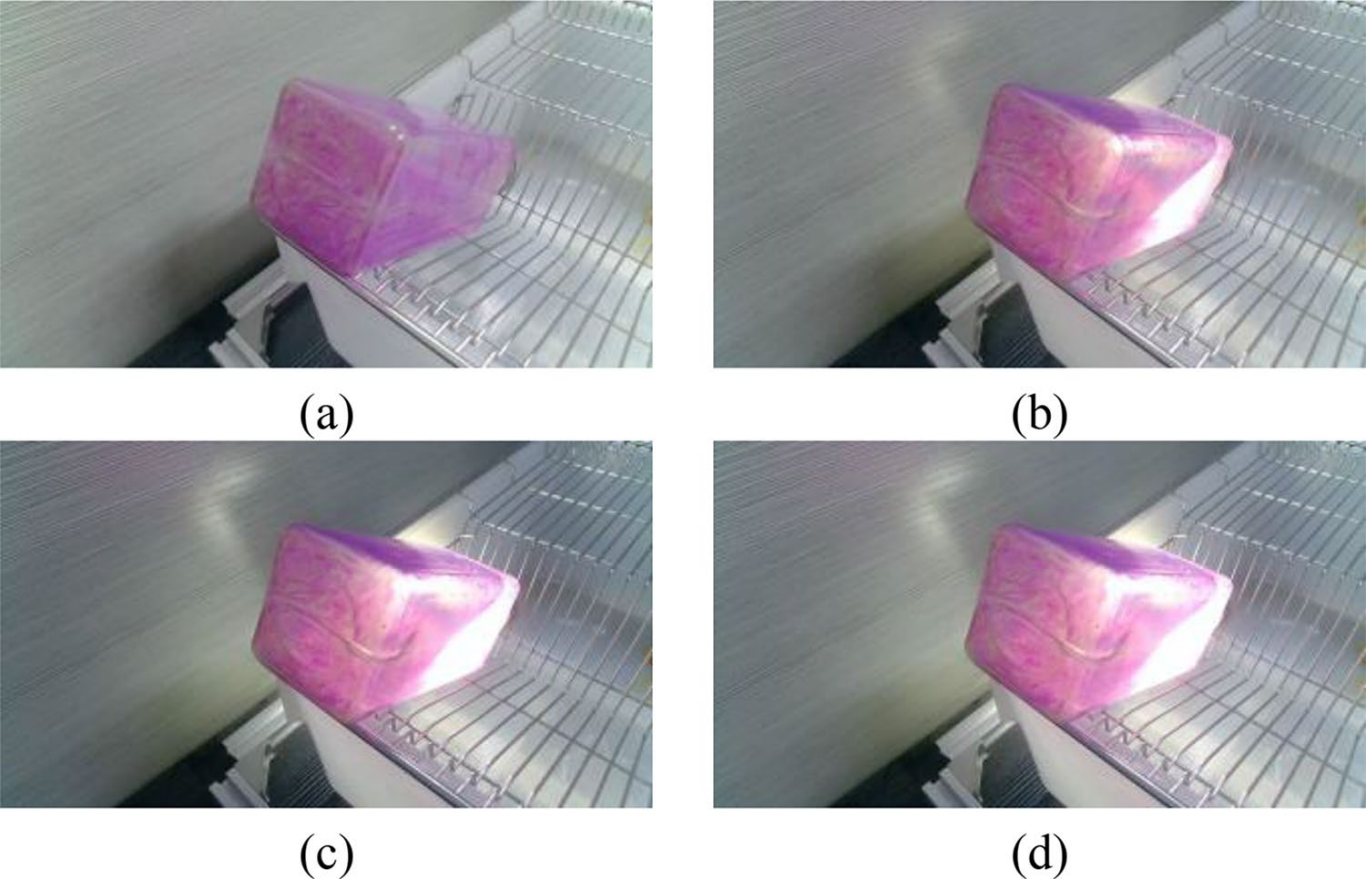
Bottles in different Illuminances (purple bottle): (**a**) 75 lx, (**b**) 91 lx, (**c**) 116 lx and (**d**) 140 lx.

In the experiment, both the light intensity and the color of the bottle will affect the extraction of the liquid level line. Therefore, in this paper, four water bottles of different colors are selected and backlit by the auxiliary light source. The influence of water bottle color and light intensity on the algorithm is tested by adjusting the illuminance. Such as Figs. 16–19

The liquid level lines of four different color bottles were extracted under 10 illumination conditions. It is evident from the image that the liquid level position is not easy to recognize in the case of natural light. After the light source is involved, the liquid level position in the image becomes apparent due to color difference.However, with the increase of light source brightness, the liquid level position becomes blurred.

**Figure 20:**
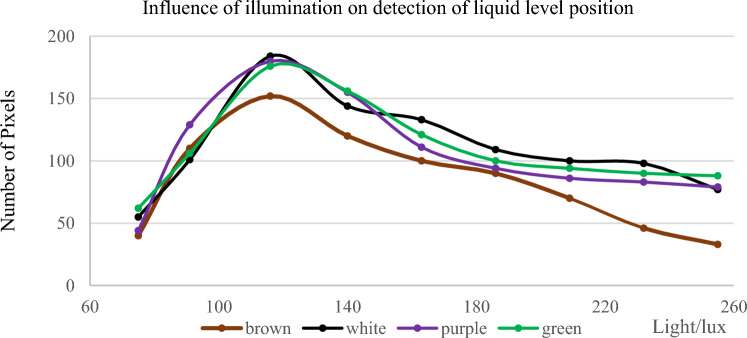
Influence of illuminance on the detection of liquid level position.

This paper takes the number of liquid level pixels detected by the Hough line as the quantitative index to quantify the detection effect. The larger the number of pixels, the better the detection effect is. Take 150 ml water and put it into a water bottle to test the number of pixels at the liquid level under four different illuminances. After the experiment, ten groups of data were obtained, as shown in Fig. 20.

When the color of the detected water bottle is different from the surrounding illuminances, the detection speed will change. Therefore, the detection speed in different cases is summarized. In Table 2, time is measured in milliseconds (ms).

**Table 2:** The detection speeds(ms).

illuminance Color	75	91	116	140	163	186	209	232	255
Green	1049	1181	1056	1075	1083	1084	1067	1125	1091
Purple	890	877	924	897	917	898	911	941	907
Brown	877	918	912	915	920	917	937	906	921
White	1359	1336	1212	1347	1316	1330	1335	1333	1317

It can be seen from Table 2, the Bottles of different colors have a specific impact on the detection speed . And the illuminance does not affect the detection speed.

According to Fig. 20, there is a specific relationship between illuminance and detection accuracy. Under the condition of specific illumination assistance, the accuracy of the detected liquid level position is relatively clear. However, with the increase of brightness, the detection accuracy decreases gradually. Because the size of the brown bottle is different from that of the other three-color bottles, the length of the liquid level will change under the same volume of water, which will lead to a great change in the number of pixels on the liquid level. The experimental results show that the effect of the liquid level line extraction algorithm is affected by the color of the bottle and the illumination intensity. The algorithm performs best under the medium illumination condition. In different applications, the effect of the liquid level line extraction algorithm can be enhanced by changing the color of the water bottle.

### Algorithm performance evaluation

In order to objectively evaluate the performance of the algorithm, the volume of 276 samples was measured by this algorithm, and were compared with the actual measurements with digital weighing balance, respectively.

The experiment involved four different colors of water bottles: white, brown, green, and purple. Based on the different colors of the water bottles, the experiment was divided into four groups, each consisting of 24 samples (a large amount of water), 24 samples (a medium amount of water), and 21 samples (a small amount of water). The experimental results are shown in Table 3.

**Table 3:** Measurement results of water volume in different color water bottles using algorithms.

Evaluating indicator	White water bottle	Purple water bottle	Green water bottle	Brown water bottle
*MAPE* (a large of water)	1.24%	2.83%	4.07%	5.80%
${R}^{2}$(a large amount of water)	0.9872	0.9418	0.8487	0.6769
*MAPE* (a medium of water)	4.09%	5.43%	7.55%	9.54%
${R}^{2}$(a medium amount of water)	0.9932	0.8706	0.7044	0.7404
*MAPE* (a small of water)	12.73%	14.29%	15.50%	15.76%
${R}^{2}$(a small amount of water)	0.9996	0.8020	0.7255	0.7255

The experimental data was evaluated using two evaluation indicators. The coefficient of determination (*R*^*2*^) and Mean Absolute Percentage Error (*MAPE*) are used to determine how close the measured value is to the actual value. The value of *R*^*2*^ indicates closeness between the estimated and the measured volumes where *R*^*2*^ = 1 indicates a perfect correlation and *R*^2^ value close to zero indicates poor correlation. The value of *MAPE* represents the error between the Estimated volume and the actual volume. The lower the value, the closer the Estimated volume is to the actual volume.

The experimental results show that the algorithm has the best measurement accuracy for the volume of water in white water bottles, while the accuracy for the volume of water in brown water bottles is the worst. This is because the color of brown water bottles has the most significant interference on the linear detection effect, which affects the accuracy of subsequent algorithms. The following text takes the white water bottle with the best algorithm measurement effect as an example to analyze the impact of water volume on detection accuracy.

**Figure 21:**
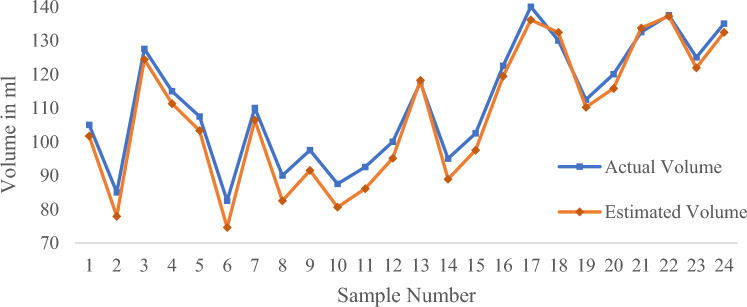
Comparison of measured and actual volume of samples (a large amount of water).

**Figure 22:**
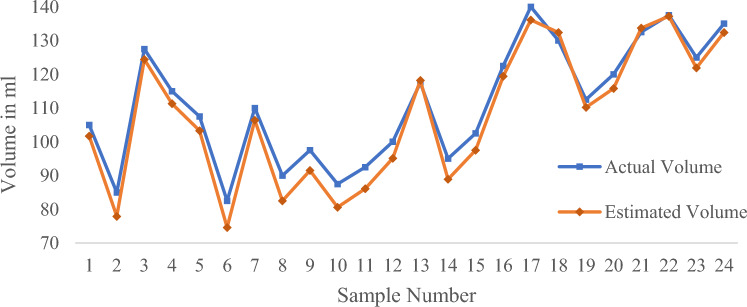
Comparison of measured and actual volume of samples (a medium amount of water).

**Figure 23:**
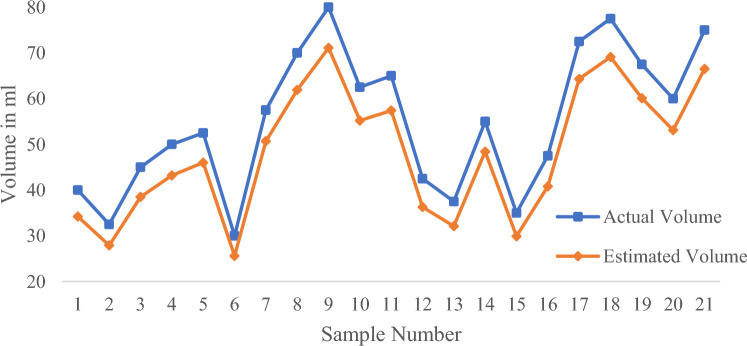
Comparison of measured and actual volume of samples (a small amount of water).

It can be seen from Figs. 21, 22, 23 and Table 3 that *R*^2^ = 0.9872 and *MAPE* = 1.24% in case of large water volume and *R*^2^ = 0.9932 and *MAPE* = 4.09% in case of medium water volume. The measured value is very close to the actual value, and the algorithm performs well. When the water volume is low, *R*^2^ = 0.9996, *MAPE* = 12.73%. Under these circumstances, the difference between the measured and actual values is significant. The accuracy of prediction results under low water volume is improved by establishing a linear regression model. Forty-eight samples were used for training purposes, and the regression equation with measured volume as the dependent variable and estimated volume as the independent variable was obtained.

24 $$\begin{array}{c}y=1.08560921x+2.66846666\end{array}$$

**Figure 24:**
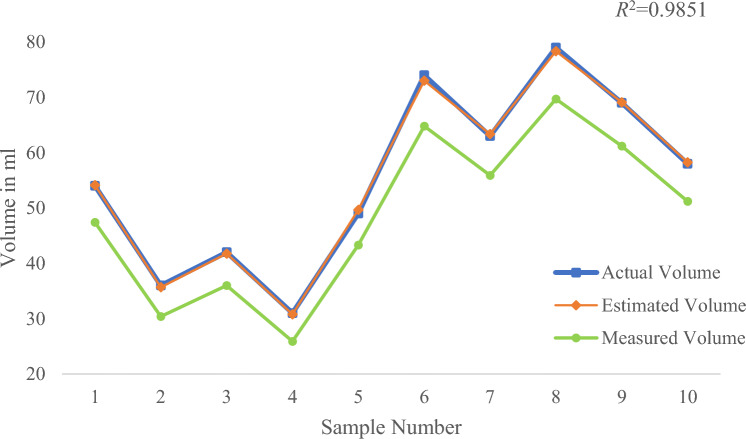
Comparison of measured, estimated and actual volume of samples (a small amount of water).

**Figure 25:**
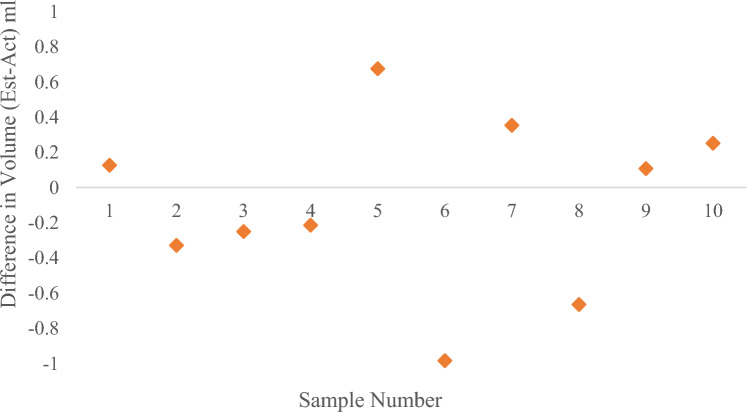
The difference in volume (estimated volume–actual volume) of samples (a small amount of water).

The developed model is used to test the data of 10 samples under low water volume, and the results are shown in Fig. 24, Fig. 25, and Table 4. *R*^2^ = 0.9996 and *MAPE* = 0.53% in case of small water volume. The model has a good prediction effect under low water conditions.

**Table 4:** Estimated and actual values of volume of sample with % deviation.

Sample no	Est. vol	Act. vol	% Dev	Sample no	Est. vol	Act. vol	% Dev
1	54.1	54	+ 0.2	6	74	73.0	+ 1.3
2	35.7	36	− 0.9	7	63	63.4	− 0.6
3	41.8	42	− 0.6	8	79	78.3	+ 0.8
4	30.8	31	− 0.7	9	69	69.1	− 0.2
5	49.7	49	+ 1.4	10	58	58.3	− 0.4
*MAPE*			0.7				

The experimental results show that the average measurement accuracy of the algorithm is above 95% under the condition of high and medium water volume. Moreover, the difference between measured volume and actual volume is relatively significant under the condition of low water volume. The reason may be that when the amount of water is small, the shape of the liquid level line at the bottle mouth is irregular. The volume of liquid at the mouth of the bottle cannot be measured, which has a particular impact on the accuracy of straight-line detection. After using linear regression model correction, the algorithm performs well in low water conditions. On the whole, the measurement accuracy of the algorithm can meet the requirements of practical use.

## Limitation

There may be some possible limitations in this study. Although this paper determined the optimal lighting intensity in the experiment, there was no specific study on the possible impact of lighting intensity on the accuracy of the algorithm. In addition, different shapes of water bottles can also affect the actual measurement performance of the algorithm. Further research can be conducted on the impact of different lighting intensities and water bottle shapes on the algorithm.

## Conclusion

This paper describes an image processing-based technique used to measure the volume of residual water in the drinking water bottle for the laboratory mouse. First, the Grabcut algorithm was used to separate the foreground from the background, and the image of the drinking bottle was extracted. The Canny edge and Hough probability line detection were then used to detect the water bottle edge and liquid level positions. Based on the projection principle, the actual length of the upper line segment of the picture is calculated, and then the volume of water is calculated. By comparing the light sources at different positions, the illuminance and the bottle with different colors, and by detecting the speed and the number of pixels of liquid level, the optimal position, the optimal illuminance, and the bottle with the best color were obtained. Finally, a linear regression model corrects the volume measurement results under low water volume to ensure that the algorithm has excellent performance under different water volumes.

It can be seen from the experimental results that according to the comparison between the actual value and the measured value, the average deviation rate is less than 5%. It means that the algorithm proposed in this paper has high accuracy in estimating the residual water volume in a square water bottle. Compared with traditional manual measurement, the method proposed in this paper has the advantage of less work and improved feeding efficiency. It can accurately estimate the amount of water in the bottle with high accuracy, improve breeding efficiency, and can meet the detection needs of large-scale breeding of laboratory mice.

## Data Availability

The data used to support the findings of this study are included within the paper.
